# Targeting lactate metabolism to restore immune homeostasis: a precision therapeutic paradigm for rheumatoid arthritis

**DOI:** 10.3389/fimmu.2025.1696932

**Published:** 2025-10-23

**Authors:** Lin Zheng, Xinyu A, Shiyu Ma, Bo Xu, Zheng Xiang, Boran Cao, Jun Shen, Xirui Xu, Yiwei Cheng, Lei Ran, Qi Shi, Yanqin Bian, Lianbo Xiao

**Affiliations:** 1 Guanghua Hospital Affiliated to Shanghai University of Traditional Chinese Medicine, Shanghai, China; 2 The Research Institute for Joint Diseases, Shanghai Academy of Traditional Chinese Medicine, Shanghai, China; 3 The Department of Pharmacy Ruijin Hospital Affiliated to Shanghai Jiaotong University School of Medicine, Shanghai, China

**Keywords:** lactate metabolism, rheumatoid arthritis, immune homeostasis, precision therapy, lactylation, metabolic reprogramming

## Abstract

The accumulation of lactate in synovial microenvironment of rheumatoid arthritis (RA) patients is closely correlated with disease activity, with elevated lactate levels serving as both biomarkers and pathogenic mediators in RA progression. In recent years, mounting evidence has demonstrated that lactate-driven metabolic reprogramming constitutes the central hub of the immune-metabolic-bone destruction axis in RA. The accumulation of lactate in the synovial microenvironment triggers a cascade of immunoregulatory effects, and these metabolic alterations create a self-perpetuating cycle of inflammation and tissue destruction, which represents the fundamental cause of RA chronicity. Therefore, lactate metabolism has been identified as a therapeutic target, opening new avenues for precision medicine approaches in RA treatment. Novel therapeutic strategies targeting lactate dehydrogenase A (LDHA) inhibitors, monocarboxylate transporters (MCTs), and enzymes that regulate lactylation have shown promising preclinical results. Furthermore, combination therapies pairing metabolic modulators with conventional biologics demonstrate synergistic effects in suppressing inflammatory pathways while improving drug penetration into synovial tissues. Additionally, patient stratification based on lactate metabolic profiles enables personalized treatment approaches. In summary, targeting lactate metabolism represents a paradigm shift in RA treatment from symptom management toward metabolic restoration. The integration of metabolic modulators with existing therapeutic regimens holds promise for achieving sustained remission and preventing long-term joint damage.

## 1 Introduction

Rheumatoid arthritis (RA) is one of the most prevalent and debilitating autoimmune diseases globally, affecting approximately 0.5-1% of the population worldwide and imposing substantial socioeconomic burden on healthcare systems (1). Despite significant advances in therapeutic interventions over the past decades, including the introduction of biologics and targeted synthetic disease-modifying antirheumatic drugs (DMARDs), a considerable proportion of patients still experience inadequate treatment responses, develop drug resistance, and suffer from serious adverse effects including increased infection risk (2). Traditional synthetic DMARDs typically take weeks to months to fully exert their therapeutic effects. For patients with high disease activity, this “window period” means inflammation may not be controlled in a timely manner, leading to the risk of continued joint damage (3). A study of 2025 found that RA patients treated exclusively with DMARDs, such as methotrexate, had greater reductions in the number of “recent thymic emigrant” (RTE) T cells, which are associated with thymic function, suggesting that the drugs may exacerbate age-related decline in the immune system rather than improve it (4). Despite the significant overall efficacy of biologics (such as TNF-α inhibitors and IL-6 inhibitors), approximately 30%-40% of patients may not respond well to their initially selected biologic. Currently, there is a lack of reliable biomarkers to accurately predict which biologic is most effective for which patient, resulting in a trial-and-error process in the early stages of treatment, delaying the optimal treatment opportunity (4). Furthermore, managing biologics is a challenge for RA patients undergoing surgery. A review of international clinical guidelines found significant disagreement regarding when biologics should be discontinued before surgery, with all recommendations based on low-quality evidence, particularly for non-orthopedic or emergency surgeries. This creates decision-making difficulties for clinicians and patients (5). For small molecule targeted drugs (tsDMARDs), such as JAK inhibitors, although randomized controlled trials (RCTs) have demonstrated the short- to medium-term safety of JAK inhibitors, studies in RA patients with cardiovascular risk factors suggest that tofacitinib (a JAK inhibitor) is associated with a higher risk of malignancies and major adverse cardiovascular events (MACE) compared with TNF inhibitors. A 5-year real-world follow-up study also pointed out the need for continued attention to the long-term safety of JAK inhibitors. Although the control of RA disease activity has improved over the past 20 years, the functional improvement of patients (assessed by the Health Assessment Questionnaire Disability Index (HAQ-DI)) has not yet been fully met (6).The heterogeneity of RA, coupled with its pathophysiology involving complex interactions between genetic susceptibility, environmental triggers, and immune dysregulation, underscores the urgent need for developing novel therapeutic paradigms that address the fundamental mechanisms driving disease persistence and progression.

Traditional understanding of RA pathogenesis has primarily focused on immune system dysfunction, characterized by the breakdown of self-tolerance, activation of autoreactive T and B cells, and production of pathogenic autoantibodies such as rheumatoid factor (RF) and anti-citrullinated protein antibodies (ACPAs) (7). However, emerging evidence from metabolomics and immunometabolism research indicates that metabolic reprogramming represents a fundamental and previously underappreciated driving factor in RA pathogenesis (8). The inflamed synovial tissue in RA joints exhibits a distinctive metabolic signature characterized by enhanced aerobic glycolysis, similar to the Warburg effect observed in cancer cells, leading to substantial lactate accumulation in synovial fluid and surrounding tissues (9). Recent breakthrough studies demonstrating the correlation between synovial fluid lactate levels and disease activity scores, including the 28-joint Disease Activity Score (DAS28), have established lactate as a reliable biomarker for disease monitoring and a potential therapeutic target (10). Recent discoveries in the field of lactylation, a novel post-translational modification involving the covalent attachment of lactyl groups to lysine residues on histones and other proteins, have provided crucial insights into the molecular mechanisms underlying lactate’s pleiotropic effects on gene expression and cellular function (11).

The identification of lactate as a central mediator in RA pathogenesis has profound implications for therapeutic intervention. Unlike traditional approaches that target individual inflammatory pathways or immune cell subsets, lactate-targeting therapies promise to simultaneously modulate multiple aspects of the disease process, including metabolic dysfunction, immune dysregulation, and tissue destruction. This comprehensive approach aligns with the growing consensus that effective RA treatment requires addressing the complex interplay between metabolism and immunity, rather than focusing solely on immunosuppression (12). The purpose of this comprehensive review is to systematically analyze current understanding of lactate metabolism’s role in RA pathogenesis and evaluate the therapeutic potential of lactate-targeting interventions. We will examine the molecular mechanisms of lactate-driven immune dysfunction, assess preclinical and clinical evidence supporting lactate-based therapeutic strategies, and propose precision medicine approaches for patient stratification and treatment selection. Furthermore, we will discuss the challenges and opportunities in translating lactate metabolism research into clinical practice, with particular emphasis on combination therapeutic strategies and personalized treatment algorithms.

## 2 Lactate metabolic reprogramming in rheumatoid arthritis

### 2.1 Aerobic glycolysis in synovial fibroblasts

Compared to healthy synovial tissue, the metabolic environment of RA joints undergoes fundamental alterations, with synovial fibroblasts experiencing dramatic metabolic transformation similar to the Warburg effect^1^ observed in cancer cells (13). However, unlike the classical Warburg effect where cancer cells preferentially utilize glycolysis to meet their own energy demands, RA synovial fibroblasts (RASFs) exhibit what is termed the “reverse Warburg effect” wherein these cells function as metabolic factories, producing and secreting lactate to fuel neighboring immune cells (14).

This metabolic reprogramming in RASFs is triggered by the hypoxic microenvironment characteristic of inflamed synovial tissue. Tissue oxygen levels in RA joints can drop to 1-2%, compared to 7-9% in healthy synovium, creating a chronic hypoxic state that triggers activation of hypoxia-inducible factor-1α (HIF-1α) (15), Ultimately upregulating key glycolytic enzymes such as hexokinase 2 (HK2), pyruvate kinase M2 (PKM2), and lactate dehydrogenase A (LDHA) (16). In RASFs, PKM2 expression is significantly elevated compared to osteoarthritis synovial fibroblasts, and its activity is modulated by inflammatory cytokines such as tumor necrosis factor-α (TNF-α) and interleukin-1β (IL-1β) (17). LDHA expression in RASFs is markedly higher than in normal synovial fibroblasts, and this upregulation correlates with disease severity and inflammatory burden (18).The metabolic transformation of RASFs extends beyond enhanced glycolysis to include alterations in mitochondrial function and oxidative metabolism. Although RASFs retain some capacity for oxidative phosphorylation, they exhibit decreased mitochondrial respiratory capacity and increased reactive oxygen species (ROS) production (19). This mitochondrial dysfunction contributes to overall metabolic stress within synovial tissue and may play a role in the resistance of RASFs to apoptosis, a characteristic that contributes to synovial hyperplasia and pannus formation. The reverse Warburg effect in RASFs establishes a metabolic symbiosis with infiltrating immune cells, particularly macrophages and T cells, which have high energy demands for proliferation, activation, and effector functions. This metabolic network transforms synovial tissue into a self-sustaining inflammatory microenvironment that can persist even in the absence of ongoing antigenic stimulation (20).

### 2.2 Lactate transport and sensing mechanisms

The biological effects of lactate in RA pathogenesis critically depend on efficient transport mechanisms that regulate lactate distribution within the synovial microenvironment and its uptake by target cells. Monocarboxylate transporters (MCTs) represent the primary mechanism for lactate transport across cellular membranes, with the MCT family comprising four major subtypes (MCT1-4) (21). MCT1 exhibits high affinity for lactate (Km ~3-5mM) and bidirectional transport capacity, being widely expressed across multiple cell types within synovial tissue, including endothelial cells, some immune cell subsets, and synovial fibroblasts (22). In the context of RA, MCT1 expression is upregulated in response to inflammatory stimuli, facilitating increased intercellular lactate flux and contributing to the establishment of lactate gradients within tissues. In contrast, MCT4 has lower affinity for lactate (Km ~15-20mM) but higher transport capacity, making it particularly suited for lactate export from highly glycolytic cells (23). The upregulation of MCT4 in RA synovial tissue has been consistently observed and correlates with disease activity, suggesting its potential utility as both a biomarker and therapeutic target (24).

Beyond passive transport, lactate sensing mechanisms play crucial roles in mediating the biological effects of elevated lactate concentrations. G-protein coupled receptor 81 (GPR81) serves as the primary lactate-sensing receptor in mammalian cells (25). Lactate activation of GPR81 leads to inhibition of adenylyl cyclase and subsequent reduction in cyclic adenosine monophosphate (cAMP) levels, resulting in decreased protein kinase A (PKA) activity (26). This signaling cascade has profound effects on immune cell function, including suppression of pro-inflammatory cytokine production in macrophages and modulation of T cell activation and differentiation. Recent studies have identified GPR81 as a key mediator of lactate-induced immunosuppression, with GPR81 knockout mice exhibiting enhanced inflammatory responses and reduced sensitivity to lactate-mediated immune suppression (27).

The spatial distribution of MCTs and GPR81 within RA synovial tissue creates distinct microenvironments with varying lactate concentrations and signaling activities. Regions with high MCT4 expression are often associated with metabolically active RASFs and inflammatory infiltrates, acting as a source of lactate, while regions with high MCT1 expression act as lactate confluence sensing zones, synergistically leading to elevated local lactate levels. High GPR81 expression amplifies the signaling from lactate, ultimately leading to further increases in lactate concentrations in the microenvironment and promoting the growth of RASFs (28). This spatial heterogeneity results in complex and sometimes contradictory effects of lactate on different immune cell populations within the same tissue.

### 2.3 Lactylation: a novel post-translational modification

The discovery of lactylation as a novel post-translational modification has revolutionized our understanding of how lactate exerts biological effects beyond its role as a metabolic substrate. In the context of RA pathogenesis, this modification has emerged as a critical mediator of lactate’s effects on gene expression and cellular function (11). The first step in the biochemical pathway for protein lactylation is the conversion of lactate to lactyl-CoA, particularly acyl-CoA synthetase short-chain family member 2 (ACSS2) (29). Lactyl-CoA then serves as the donor molecule for lactylation reactions, which can occur through both enzymatic and non-enzymatic mechanisms. While specific lactyltransferases have not been definitively identified, several acetyltransferases, including p300/CBP and PCAF, have been shown to possess lactyltransferase activity under certain conditions (30).

The regulation of lactylation is achieved through the action of delactylases, primarily the NAD+-dependent deacetylases of the sirtuin family. SIRT1, SIRT2, and SIRT3 have all been shown to possess delactylase activity, with SIRT3 being particularly important for regulating mitochondrial protein lactylation (31). The balance between lactylation and delactylation is influenced by cellular NAD+ levels, lactate concentrations, and cellular metabolic state, providing multiple regulatory points for this modification.

In the context of RA, lactylation plays a crucial role in regulating immune cell function and inflammatory responses. Studies using single-cell RNA sequencing and chromatin immunoprecipitation have revealed that plasma cells from RA patients exhibit the highest lactylation scores among all immune cell types, with significant upregulation of lactylation-related genes (32). The functional consequences of lactylation extend beyond histone modifications to include lactylation of non-histone proteins involved in metabolic regulation, signal transduction, and cellular stress responses. Proteomic analyses have identified hundreds of lactylated proteins in RA synovial tissue, including key enzymes in glycolysis, the TCA cycle, and oxidative phosphorylation (33). Lactylation of these metabolic enzymes can alter their activity, stability, and subcellular localization, creating feedback loops that further amplify metabolic dysfunction. The therapeutic implications of lactylation in RA are significant, as this modification represents a druggable target that directly links metabolism to gene expression. Small molecule inhibitors of lactylation, including SIRT activators and lactyl-CoA synthesis inhibitors, have shown promise in preclinical models of inflammatory diseases (34).

## 3 Lactate-driven disruption of immune homeostasis

The effects of lactate on the function of T cells and myeloid cells play a central role in the initiation and maintenance of inflammatory responses. The functional effects of lactate depend on its concentration, cell type specificity, and the corresponding physiological or pathological state. The high lactate concentration unique to RA synovial tissue profoundly alters the differentiation, activation, and effector function of T cells (35). Furthermore, the lactate-rich microenvironment of RA joints profoundly alters the function of myeloid cells, leading to persistent inflammation, impaired tissue repair, and enhanced bone and cartilage destruction (36).

### 3.1 T cell polarization dysfunction

The impact of lactate on T cell function represents one of the most significant and complex aspects of lactate-mediated immune dysfunction in RA. As central orchestrators of adaptive immune responses, T cells are exquisitely sensitive to metabolic cues in their microenvironment, and the high lactate concentrations characteristic of RA synovial tissue profoundly alter T cell differentiation, activation, and effector functions (35).

#### 3.1.1 Th17 cell dysfunction and metabolic reprogramming

Th17 cells, characterized by their production of interleukin-17A (IL-17A), IL-17F, and IL-22, play a central role in RA pathogenesis through their ability to promote synovial inflammation, recruit neutrophils, and stimulate the production of matrix metalloproteinases (MMPs) that contribute to cartilage and bone destruction (37). Under physiological conditions, Th17 cell differentiation and function are heavily dependent on glycolytic metabolism, with these cells exhibiting high rates of glucose uptake and lactate production to meet their energy and biosynthetic demands (38). However, in the lactate-rich environment of RA joints, Th17 cells undergo a paradoxical metabolic and functional reprogramming that ultimately leads to their dysfunction. Exposure to elevated extracellular lactate concentrations (10–25 mM) triggers a comprehensive metabolic rewiring in Th17 cells, characterized by a shift from glycolysis toward oxidative phosphorylation and a significant reduction in lactate production (39). This metabolic transition is accompanied by dramatic changes in gene expression, with downregulation of Th17-specific transcription factors such as RORγt and STAT3, and corresponding decreases in IL-17A and IL-17F production.

The mechanism underlying lactate-induced Th17 dysfunction involves multiple interconnected pathways. First, lactate uptake through MCT1 leads to intracellular acidification and metabolic stress, triggering the activation of AMP-activated protein kinase (AMPK) and subsequent inhibition of mTORC1 signaling (40). Since mTORC1 is essential for Th17 cell activation and IL-17 production, its inhibition by lactate effectively suppresses Th17 effector function. Second, lactate-induced changes in cellular redox state, particularly the accumulation of reactive oxygen species (ROS), activate stress response pathways that interfere with Th17 cell survival and function (41).Perhaps most importantly, lactate exposure leads to extensive histone lactylation in Th17 cells, particularly at the H3K18 position, which directly impacts the transcriptional regulation of Th17-associated genes (42).

#### 3.1.2 Regulatory T cell impairment

Regulatory T cells (Tregs), characterized by the expression of the transcription factor Foxp3 and their ability to suppress immune responses, play crucial roles in maintaining immune homeostasis and preventing autoimmune disease. In RA, Treg function is compromised, contributing to the breakdown of self-tolerance and the persistence of inflammatory responses. The lactate-rich synovial microenvironment significantly contributes to Treg dysfunction through multiple mechanisms that impair both Treg development and suppressive function (43).

The relationship between lactate and Treg function is complex and context-dependent. While some studies have suggested that lactate can promote Treg differentiation under certain conditions, the chronic exposure to high lactate concentrations characteristic of RA appears to have predominantly detrimental effects on Treg function (44). The primary mechanism involves lactate-induced modulation of Foxp3 expression and stability through epigenetic modifications and post-translational regulation. SIRT1, a NAD+-dependent deacetylase that plays crucial roles in Treg function, is significantly affected by the altered metabolic environment in lactate-exposed cells. High lactate concentrations lead to changes in the NAD+/NADH ratio and cellular energy status that impair SIRT1 activity. Since SIRT1 is required for the deacetylation and stabilization of Foxp3, its inhibition in lactate-rich environments leads to Foxp3 degradation and loss of Treg suppressive function (45).

Furthermore, lactate exposure alters the metabolic programming of Tregs, shifting them away from their preferred oxidative metabolism toward glycolysis. This metabolic reprogramming is incompatible with optimal Treg function, as these cells require efficient oxidative phosphorylation and fatty acid oxidation to maintain their suppressive capabilities and long-term survival. The metabolic stress induced by forced glycolytic metabolism in Tregs leads to increased apoptosis and reduced suppressive capacity (46).

#### 3.1.3 CD8+ T cell and other T cell subset effects

While much attention has focused on CD4+ T cell subsets, lactate also significantly affects CD8+ T cells and other T cell populations in RA. Lactate exposure has been shown to impair CD8+ T cell activation and effector function through mechanisms similar to those observed in CD4+ T cells, including metabolic reprogramming, epigenetic modifications, and altered signaling pathways (47). The effects of lactate on other T cell subsets, including γδ T cells, natural killer T (NKT) cells, and mucosal-associated invariant T (MAIT) cells, are less well characterized but likely contribute to the overall immune dysfunction observed in RA (36).

### 3.2 Myeloid cell dysfunction

Myeloid cells, including macrophages, dendritic cells, and neutrophils, represent the most abundant immune cell populations in RA synovial tissue and play central roles in both the initiation and perpetuation of inflammatory responses (48). The lactate-rich microenvironment of RA joints profoundly alters myeloid cell function, leading to sustained inflammation, impaired tissue repair, and enhanced bone and cartilage destruction.

#### 3.2.1 Macrophage polarization and metabolic reprogramming

Macrophages exhibit remarkable plasticity in their activation states, with the classical M1 (pro-inflammatory) and alternative M2 (anti-inflammatory/tissue repair) polarization states representing the extremes of a functional spectrum. In healthy tissues, the balance between M1 and M2 macrophages is tightly regulated to ensure appropriate inflammatory responses followed by resolution and tissue repair (49). However, in RA, this balance is disrupted, with persistent M1 activation and impaired M2 function contributing to chronic inflammation.

Lactate has emerged as a critical regulator of macrophage polarization, with the capacity to promote M2 polarization while suppressing M1 responses under certain conditions (50). However, the effects of lactate on macrophage function in RA are complex and depend on multiple factors, including lactate concentration, duration of exposure, and the presence of other inflammatory mediators.At moderate concentrations (5–15 mM), lactate can promote M2 macrophage polarization through several mechanisms. First, lactate serves as an alternative carbon source for the tricarboxylic acid (TCA) cycle, supporting the oxidative metabolism that is characteristic of M2 macrophages. The utilization of lactate for oxidative phosphorylation provides the energy necessary for M2 effector functions, including the production of anti-inflammatory cytokines such as IL-10 and TGF-β, and the synthesis of extracellular matrix components involved in tissue repair (51). Second, lactate-induced histone lactylation directly promotes the transcription of M2-associated genes. ChIP-seq analyses have revealed that H3K18 lactylation is enriched at the promoters of key M2 genes, including Arg1 (arginase 1), Ccl22 (C-C motif chemokine ligand 22), and Il10 (52). The presence of lactylation marks at these loci is associated with increased chromatin accessibility and enhanced transcriptional activity, providing a direct epigenetic mechanism for lactate-induced M2 polarization.

However, at the high lactate concentrations characteristic of RA synovial fluid (20–40 mM), the effects on macrophages become more complex and potentially detrimental. Chronic exposure to very high lactate concentrations can lead to metabolic stress, mitochondrial dysfunction, and impaired macrophage function. Under these conditions, macrophages may exhibit features of both M1 and M2 activation, resulting in a dysfunctional phenotype that contributes to persistent inflammation and tissue damage (53).

#### 3.2.2 Dendritic cell dysfunction

Dendritic cells (DCs) serve as professional antigen-presenting cells that bridge innate and adaptive immunity through their ability to capture, process, and present antigens to T cells. In RA, DCs play crucial roles in the presentation of self-antigens and the activation of autoreactive T cells, contributing to the breakdown of immune tolerance and the initiation of autoimmune responses (54).

Lactate exposure significantly impairs DC function through multiple mechanisms. First, lactate interferes with DC maturation and activation, leading to reduced expression of co-stimulatory molecules such as CD80, CD86, and CD40, and decreased production of pro-inflammatory cytokines such as IL-12 and TNF-α (55). This impairment in DC activation capacity reduces their ability to effectively prime T cell responses and may contribute to the development of immune tolerance. Second, lactate alters DC metabolism, shifting these cells away from the glycolytic metabolism required for optimal activation toward oxidative phosphorylation (56). While this metabolic shift might seem beneficial, it actually impairs DC function because activated DCs require rapid ATP production through glycolysis to support their energy-intensive processes, including antigen processing, migration, and cytokine production. Third, lactate-induced lactylation in DCs affects the expression of genes involved in antigen presentation and T cell activation. Proteomic analyses have revealed extensive lactylation of proteins involved in MHC class II antigen presentation, including components of the peptide loading complex and regulatory proteins that control MHC expression. The lactylation of these proteins can alter their function and stability, leading to impaired antigen presentation capacity (57).

#### 3.2.3 Neutrophil function and NETosis

Neutrophils are among the first immune cells recruited to sites of inflammation and play important roles in RA pathogenesis through their release of inflammatory mediators, proteases, and neutrophil extracellular traps (NETs) The formation of NETs, a process known as NETosis, involves the release of chromatin decorated with antimicrobial proteins and has been implicated in the generation of autoantibodies and the perpetuation of inflammatory responses in RA (58).

Lactate has complex effects on neutrophil function, with both stimulatory and inhibitory effects depending on concentration and context. At moderate concentrations, lactate can enhance neutrophil activation and NET formation, potentially contributing to tissue damage and autoantibody production. However, at very high concentrations, lactate may impair neutrophil function and survival, leading to defective bacterial clearance and increased susceptibility to infections (59).

The metabolic effects of lactate on neutrophils are particularly important given their heavy reliance on glycolytic metabolism. Neutrophils have limited mitochondrial capacity and depend primarily on glycolysis for ATP production. Lactate exposure can interfere with glycolytic flux and energy production in neutrophils, leading to impaired effector functions and reduced survival (60).

Recent studies have also identified lactate as a modulator of NET formation through its effects on histone modifications and chromatin structure. The lactylation of histones in neutrophils can alter chromatin accessibility and may influence the propensity for NET formation (61). (**Table 1**).

**Table 1:** Lactate-driven disruption of immune homeostasis.

Type	Name	Functional impact	Reference
T cells	Th17 cells	Characterized by the production of interleukin-17A (IL-17A), IL-17F, and IL-22, leading to synovial inflammation	Miossec P, et al (37)
	Treg	Leading to the breakdown of self-tolerance and the continuation of inflammatory response	Sakaguchi S, et al (43)
	CD4+ T cells	Causes tissue damage through direct cytotoxicity and inflammatory cytokine production	Rundqvist H, et al (47)
	CD8+ T cells	Impairment of CD8+ T cell activation and effector function	Khatib-Massalha E, et al (48)
Myeloid cells	Macrophages	Promotes M2 polarization while inhibiting M1 responses	Shi W, et al (50)
	DCs	Interfering with DCs maturation and activation, lactate alters DC metabolism and affects the expression of genes involved in antigen presentation and T cell activation	Fang Y, et al (57)
	Neutrophils	Depending on the concentration and environment, it has both stimulatory and inhibitory effects	Borregaard N, et al (59)

## 4 Vicious cycle: lactate and ra disease progression

### 4.1 Synovitis-bone destruction axis

The synovitis-bone destruction axis encompasses the intricate relationships between inflammatory synovial tissue, cartilage degradation, and bone erosion, with lactate playing a central role in each component of this destructive cascade.

Lactate accumulation in synovial tissue directly promotes pannus formation through multiple mechanisms. First, lactate serves as a metabolic fuel for rapidly proliferating synovial fibroblasts, which constitute the bulk of the hyperplastic synovial lining (62). The availability of lactate as an alternative carbon source enables these cells to maintain high proliferation rates even under the hypoxic conditions characteristic of inflamed joints. This metabolic flexibility is crucial for the sustained growth of pannus tissue and its ability to invade adjacent cartilage and bone. Second, lactate promotes angiogenesis within synovial tissue through its effects on endothelial cell function and vascular endothelial growth factor (VEGF) expression (63). The hypoxic environment and lactate accumulation in RA synovium create powerful angiogenic stimuli that drive the formation of new blood vessels. Lactate-induced VEGF upregulation occurs through both HIF-1α-dependent and independent mechanisms (64).

In RA, cartilage degradation occurs through both direct invasion by pannus tissue and indirect mechanisms involving the release of cartilage-degrading enzymes and inflammatory mediators. Lactate contributes to cartilage destruction through its effects on chondrocyte metabolism and function. Chondrocytes, the resident cells of cartilage, normally maintain cartilage matrix through a balance of anabolic and catabolic processes. However, exposure to the high lactate concentrations present in RA synovial fluid disrupts this balance, leading to enhanced matrix degradation and impaired repair mechanisms (65). The metabolic effects of lactate on chondrocytes are particularly significant because these cells are adapted to the avascular, hypoxic environment of cartilage (66). Chondrocytes rely primarily on glycolytic metabolism for energy production and can function at very low oxygen tensions. However, the additional metabolic stress imposed by high extracellular lactate concentrations may exceed their adaptive capacity, leading to cellular dysfunction and death. Furthermore, lactate exposure induces the expression of catabolic enzymes in chondrocytes, including aggrecanases (ADAMTS-4 and ADAMTS-5) and collagenases (MMP-1 and MMP-13), while suppressing the expression of anabolic factors such as type II collagen and aggrecan (67). Recent studies have also identified lactate as a regulator of chondrocyte autophagy, with lactate exposure impairing autophagic flux in chondrocytes, leading to the accumulation of damaged organelles and proteins that contribute to cellular dysfunction and cartilage degradation (68).

**Figure 1:**
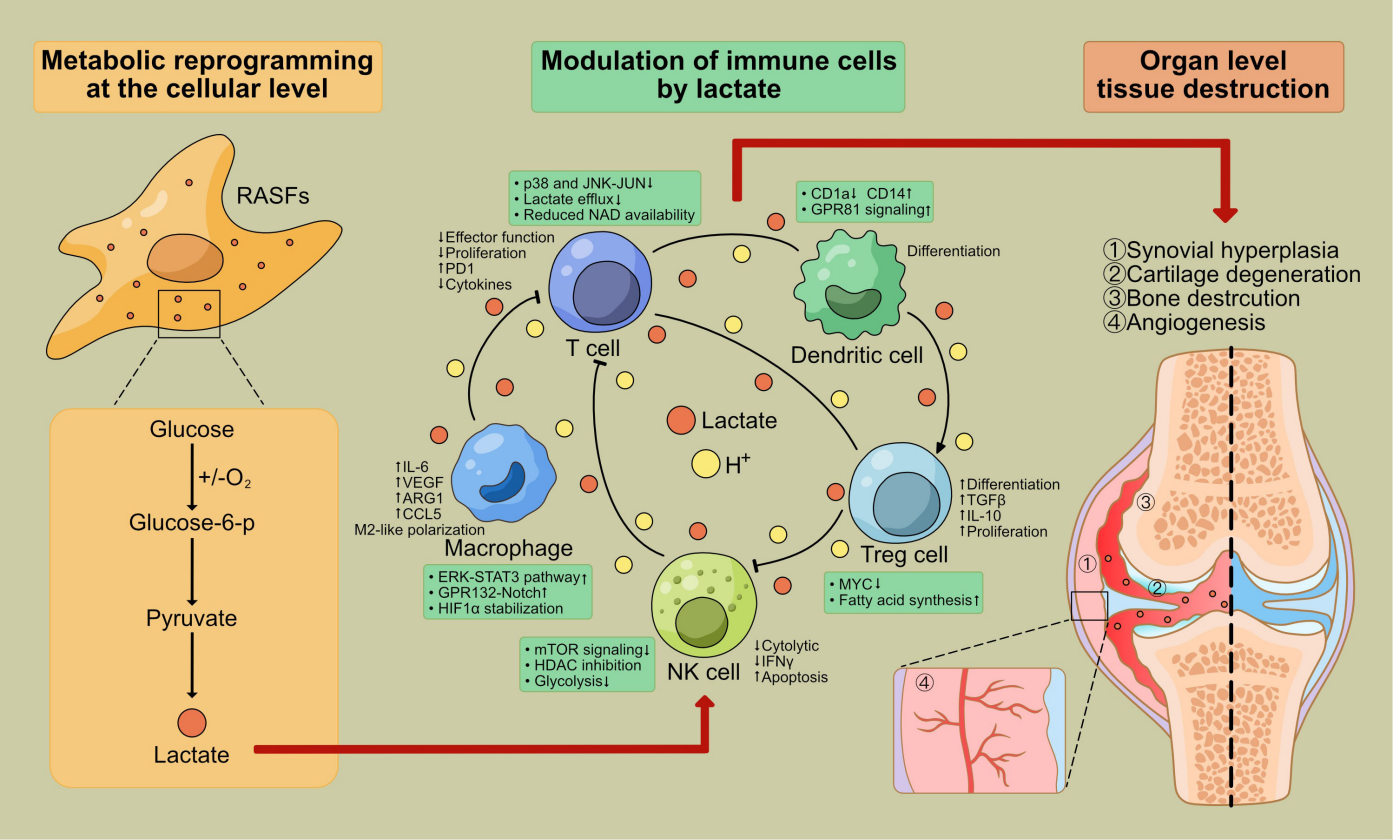
The complete pathological process of lactate from metabolic reprogramming at the cellular level to tissue destruction at the organ level. **①**Left side (metabolic reprogramming at the cellular level): The left side of the figure will focus on the enhanced glycolysis in synovial fibroblasts of RA patients and the resulting lactate accumulation. This will be the starting point of the pathological process. **②**Middle Section (Regulatory Effects of Lactate on Immune Cells): The middle section will describe the complex regulatory effects of lactate on various immune cell subsets, including immune cells, blood vessels, and cellular metabolites such as lactate. Due to the Warburg effect, lactate accumulation causes acidosis, angiogenesis, immunosuppression, and FLS proliferation and survival. **③**Right side (organ level tissue destruction): The right side of the figure will show the joint tissue destruction ultimately caused by RA, including: synovial hyperplasia, cartilage degradation, bone destruction, angiogenesis. This figure was created with reference to the cited literature [PMID: 34177945 (126)].

Lactate significantly enhances osteoclast differentiation and bone resorption activity through multiple mechanisms. First, lactate directly modulates RANKL signaling in osteoclast precursors, enhancing their responsiveness to RANKL stimulation and promoting their differentiation into mature osteoclasts (69). Second, lactate-induced lactylation in osteoclasts affects the expression of genes involved in bone resorption. Proteomic analyses have revealed extensive lactylation of proteins involved in osteoclast function, including components of the bone resorption machinery and regulatory proteins that control osteoclast activity (70). Lactylation of these proteins can enhance their function and stability, leading to increased bone resorption capacity. Additionally, the role of HIF-1α in lactate-mediated osteoclast activation has been particularly well characterized. HIF-1α stabilization in osteoclasts promotes the expression of glycolytic enzymes, including GLUT1, LDHA, and MCT4, creating a positive feedback loop that enhances lactate production and utilization (71). This metabolic reprogramming supports the high energy demands of bone resorption and contributes to sustained osteoclast activity. (**Figure 1**).

### 4.2 Therapeutic resistance mechanisms

The development of therapeutic resistance represents a major clinical challenge in RA management, with a substantial proportion of patients failing to achieve adequate disease control despite multiple treatment options (72). The lactate-rich microenvironment of RA joints contributes to therapeutic resistance through several mechanisms that impair drug efficacy and promote the persistence of inflammatory responses.

Tumor necrosis factor (TNF) inhibitors represent one of the most successful biologic therapies for RA, with drugs such as adalimumab, etanercept, and infliximab demonstrating significant efficacy in reducing inflammation and preventing joint damage. However, approximately 30-40% of patients exhibit poor responses to TNF inhibitor therapy, and many initially responsive patients eventually lose efficacy over time (73). Recent studies have identified lactate as an important contributor to TNF inhibitor resistance through its effects on cellular metabolism and drug sensitivity (74). High lactate concentrations in RA synovial tissue activate mTORC1 signaling in immune cells, leading to enhanced protein synthesis, cell survival, and anti-apoptotic responses. This metabolic reprogramming reduces the sensitivity of inflammatory cells to TNF inhibition and enables them to persist under therapeutic intervention (75). The clinical implications of lactate-mediated TNF inhibitor resistance are significant, as they suggest that combination therapies simultaneously targeting TNF and lactate metabolism may be more effective than TNF inhibition alone.

The unique tissue architecture and metabolic environment of RA synovium create significant barriers to drug penetration that can limit therapeutic efficacy (76). Lactate contributes to these drug penetration barriers through its effects on synovial tissue structure and vascular function. Lactate-induced angiogenesis promotes the formation of abnormal blood vessels with increased permeability and poor organization. While this might appear to facilitate drug delivery, the resulting tissue edema and increased interstitial pressure actually impede drug penetration into deeper tissue layers (77). Additionally, the metabolic stress induced by high lactate concentrations may impair the function of drug transporters and efflux pumps that regulate cellular drug uptake and retention. This can lead to reduced intracellular drug concentrations and diminished therapeutic effects, particularly for drugs that require active transport mechanisms for cellular uptake. The development of drug delivery systems capable of overcoming these lactate-mediated barriers represents an important area of therapeutic innovation (78).

## 5 Precision therapeutic interventions targeting lactate metabolism

Chronic inflammation in RA can lead to the development of synovial fibrosis, characterized by excessive collagen deposition and tissue scarring that further impairs joint function (79). Recent evidence suggests that lactate plays an important role in promoting fibrotic responses through its effects on fibroblast activation and extracellular matrix production. Lactate exposure induces the activation of synovial fibroblasts and their transformation into myofibroblasts, which are characterized by enhanced contractility and increased collagen production (80). The development of synovial fibrosis creates additional barriers to drug penetration and may perpetuate inflammatory responses by creating a rigid tissue environment that impairs normal joint mechanics (81). Fibrotic tissue also serves as a reservoir for inflammatory mediators that can contribute to symptom persistence even after successful control of active inflammation. Recent studies have identified potential therapeutic targets for preventing or reversing lactate-induced fibrosis. Anti-fibrotic drugs originally developed for treating pulmonary fibrosis, such as nintedanib, have shown promise in preclinical models of RA when used in combination with lactate-targeting therapies (82).

### 5.1 Small molecule inhibitors of lactate production

The development of small molecule inhibitors targeting lactate metabolism represents one of the most promising therapeutic approaches for RA (83). These compounds offer the potential to directly address the metabolic dysfunction underlying RA pathogenesis while providing opportunities for combination therapy with existing treatments.

Lactate dehydrogenase A (LDHA) represents the most direct and specific target for reducing lactate production in RA joints (84). FX-11, the most extensively studied LDHA inhibitor, exhibits potent, selective, and reversible competitive inhibition. Preclinical studies in collagen-induced arthritis (CIA) models have demonstrated that FX-11 treatment significantly reduces joint inflammation, cartilage destruction, and bone erosion (85). The therapeutic effects of FX-11 correlate with reduced synovial fluid lactate levels, decreased inflammatory cell infiltration, and improved joint function scores. The mechanism of action of FX-11 involves direct binding to the LDHA active site, preventing the conversion of pyruvate to lactate (86). GSK2837808A represents another promising LDHA inhibitor with unique pharmacological properties. This compound exhibits high selectivity for LDHA over LDHB and other related enzymes, reducing the risk of off-target effects. In preclinical studies, GSK2837808A treatment has been shown to increase hyaluronic acid production while reducing inflammatory markers, suggesting potential benefits in both inflammation control and tissue repair (87). However, the clinical development of LDHA inhibitors faces several challenges, including the need to balance efficacy with potential metabolic side effects (88). Since LDHA plays important roles in normal cellular metabolism, particularly in tissues with high glycolytic activity such as skeletal muscle and brain, careful dose optimization and patient monitoring are crucial for safe clinical use (89).

Beyond LDHA, other enzymes in the glycolytic pathway represent potential therapeutic targets for modulating lactate production (90). Hexokinase 2 (HK2), the first rate-limiting enzyme in glycolysis, is significantly upregulated in RA synovial tissue and represents an attractive target for therapeutic intervention. 2-Deoxyglucose (2-DG), a glucose analog that competitively inhibits HK2, has been extensively studied as a glycolytic inhibitor (91). While 2-DG has shown efficacy in preclinical models of inflammatory diseases, its clinical development has been limited by dose-limiting toxicity due to its effects on normal glucose metabolism. However, recent studies suggest that low-dose 2-DG therapy may provide anti-inflammatory benefits with acceptable safety profiles.

### 5.2 MCT transporter inhibition

Monocarboxylate transporters (MCTs) have emerged as attractive therapeutic targets due to their central role in lactate transport and their upregulation in RA synovial tissue (92). Inhibiting MCT function can disrupt lactate-mediated intercellular communication and alter the metabolic environment within joints.

AZD3965 is a potent and selective MCT1 inhibitor that has been extensively studied in cancer research and is now being investigated for inflammatory diseases (93). This compound exhibits high selectivity for MCT1 over other MCT subtypes and effectively blocks lactate transport across cellular membranes. In preclinical studies of arthritis, AZD3965 treatment has been shown to reduce joint inflammation and improve disease outcomes (94). The therapeutic effects correlate with altered lactate distribution within synovial tissue, reduced immune cell activation, and improved drug penetration. The ability of MCT1 inhibition to enhance drug delivery is a particularly attractive feature for combination therapeutic approaches. The mechanism of action of AZD3965 involves binding to the MCT1 transporter and preventing both lactate uptake and efflux, which disrupts the metabolic symbiosis between lactate-producing cells (such as synovial fibroblasts) and lactate-consuming cells (such as immune cells), leading to metabolic stress and reduced inflammatory activity (95).

MCT4, which is primarily responsible for lactate efflux from highly glycolytic cells, represents another important therapeutic target. Unlike the bidirectional MCT1, MCT4 functions primarily as a lactate exporter, making it particularly important for cells that produce large amounts of lactate. Antibody-based MCT4 inhibition is being investigated as a strategy to selectively target MCT4-expressing cells while avoiding effects on MCT1-dependent processes (96). Monoclonal antibodies targeting MCT4 can block lactate efflux and induce metabolic stress in highly glycolytic cells, thereby reducing inflammation and tissue damage. The development of MCT4-targeting antibodies offers several advantages, including high specificity, long half-life, and the potential for antibody-drug conjugate approaches (97). These properties make MCT4 antibodies attractive candidates for combination therapy with other anti-inflammatory drugs.

### 5.3 Lactylation modifiers

The discovery of lactylation as a key mechanism mediating lactate’s biological effects has opened new therapeutic opportunities for targeting this post-translational modification (98). Compounds capable of modulating lactylation levels promise to directly interfere with lactate-mediated gene expression changes without necessarily altering lactate metabolism.

The NAD+-dependent deacetylases of the sirtuin family, particularly SIRT1, SIRT2, and SIRT3, possess delactylase activity and represent important regulators of lactylation levels (99). Activating these enzymes can reduce pathological lactylation and restore normal gene expression patterns in inflammatory cells. Resveratrol and its analogs have been extensively studied as SIRT1 activators and have shown anti-inflammatory effects in preclinical models of arthritis. The therapeutic effects of resveratrol correlate with reduced lactylation of key inflammatory genes and improved immune cell function (100). However, the clinical development of resveratrol has been limited by poor bioavailability and rapid metabolism. SRT1720 represents a more potent and selective SIRT1 activator than resveratrol, with improved pharmacological properties (101). This compound exhibits better bioavailability and longer half-life, making it more suitable for clinical development. Preclinical studies have shown that SRT1720 treatment can reduce joint inflammation and improve disease outcomes in animal models of arthritis. Nicotinamide riboside (NR) and nicotinamide mononucleotide (NMN) are NAD+ precursors that can enhance SIRT activity by increasing cellular NAD+ levels (102). These compounds offer an alternative to direct SIRT activation and have shown promise in preclinical studies of inflammatory diseases.

### 5.4 Combination therapeutic strategies

The complexity of lactate-mediated pathways in RA suggests that combination approaches targeting multiple aspects of lactate metabolism may be more effective than single-agent therapies (103). Several combination strategies are being investigated in preclinical studies and early clinical trials.

LDHA inhibitor + JAK inhibitor represents a particularly promising combination approach (104). JAK inhibitors, such as tofacitinib and baricitinib, target key inflammatory signaling pathways, while LDHA inhibitors address the metabolic dysfunction underlying RA pathogenesis. Preclinical studies have demonstrated synergistic effects of this combination, with enhanced suppression of inflammatory cytokines and improved joint protection. The rationale for this combination lies in their complementary mechanisms of action (105). JAK inhibitors block cytokine signaling pathways that drive inflammation, while LDHA inhibitors reduce the metabolic fuel that sustains inflammatory cell function. The combination approach allows for lower doses of each drug, potentially reducing side effects while maintaining or enhancing therapeutic efficacy. MCT inhibitor + TNF inhibitor represents another promising combination strategy. MCT inhibition can improve drug penetration into synovial tissue, potentially enhancing the efficacy of TNF inhibitors. Additionally, the metabolic effects of MCT inhibition can complement the anti-inflammatory actions of TNF blockade (106). LDHA inhibitor + MCT inhibitor offers the potential to simultaneously reduce lactate production and disrupt lactate transport (107). This dual approach may more comprehensively address lactate-mediated pathology and could be particularly effective in patients with high lactate levels or extensive synovial involvement. Furthermore, glycolytic inhibitor + oxidative phosphorylation enhancer represents a strategy to simultaneously reduce lactate production and promote alternative metabolic pathways (108). Compounds that enhance mitochondrial function, such as coenzyme Q10 analogs or PGC-1α activators, can be combined with glycolytic inhibitors to promote metabolic reprogramming toward oxidative metabolism. It has been reported in the literature that diclofenac (DC, inhibiting GLUT-1 to block glucose uptake) and lonidamine (LND, inhibiting HK-2 to block glycolysis) were coupled to hyaluronic acid (HA) through a disulfide linker (cysteamine) to form a polymer-prodrug conjugate, which was co-assembled into dual-prodrug nanoparticles in a 2:1 ratio. Through targeted delivery, FLS-specific metabolic regulation was achieved, lactate-induced FLS-macrophage crosstalk was reduced, and the synovial microenvironment was improved (109) (**Table 2**).

**Table 2:** Summary of investigational drugs targeting lactate pathways.

Classification	Drug name	Target	Mechanism of action	Primary effects	Development status	Potential combination regimens
LDHA inhibitor	FX-11	LDHA	Competitive inhibition of lactate dehydrogenase A	Reduces lactate production,inhibits glycolysis	Preclinical research	MTX+FX-11,TNF inhibitor +FX-11
	GSK2837808A	LDHA	Highly selective LDHA inhibitor	Nanomolar-level inhibitory activity, improves joint inflammation	Preclinical research	JAK inhibitor combination,Biologic agent combination
MCT inhibitor	AZD3965	MCT1	Blocks lactate transport, nanomolar-level inhibition	Reduces lactate efflux, improves drug penetration	Phase I clinical trial	TNF inhibitor combination, Chemotherapy drug combination
	AR-C155858	MCT1/MCT2	Dual MCT inhibitor	Broad-spectrum lactate transport blockade	Preclinical research	Metabolic modulator combination
	VB124	MCT4	Selective MCT4 inhibitor	Targets highly glycolytic cells	Preclinical research	LDHA inhibitor combination
Lactic acid modification regulator	Resveratrol	Sirtuin activation	Enhances delactylase activity	Anti-inflammatory+ metabolic regulation	Nutritional supplement	Traditional DMARD combination
	SRT1720	SIRT1 activator	Specific SIRT1 activation	Potent delactylation activity	Preclinical research	Metabolic syndrome treatment
	Nicotinamide riboside	NAD+ precursor	Restores NAD+/NADH ratio	Improves mitochondrial function	Nutritional supplement	Anti-aging therapy combination
Indirect metabolic regulator	2-Deoxyglucose	Glycolysis inhibition	Blocks the first step of glycolysis	Comprehensive glycolysis inhibition	Preclinical research	Use with caution,high toxicity
Combination Therapy	LDHA inhibitor+ JAK inhibitor	Multi-target	Metabolic+immune dual regulation	Synergistic anti-inflammatory effects	Proof of concept	Tofacitinib/Baricitinib
	MCT inhibitor+ TNF inhibitor	Multi-target	Improved drug penetration +anti-inflammatory	Enhanced biologic agent efficacy	Proof of concept	Adalimumab/Infliximab
	Metabolic modulator+MTX	Multi-target	Metabolic reset+anti-proliferative	Enhanced traditional DMARD effects	Proof of concept	Methotrexate combination

Development Status Classification: Preclinical research-In vitro and animal model validation stage; Phase I clinical trial-Safety and pharmacokinetic assessment; Drug repositioning study-New indication research for approved drugs; Nutritional supplement-Natural compounds that can serve as adjuvant therapy; Proof of concept-Preliminary validation of combination therapy strategies.

### 5.5 Patient stratification and precision medicine

The heterogeneity of RA patients necessitates personalized approaches to lactate-targeting therapies (110). Patient stratification based on metabolic profiles, genetic factors, and biomarker expression can help identify individuals most likely to benefit from specific therapeutic interventions.

High lactate phenotype patients (serum lactate >4mM) represent a distinct subgroup that may be particularly responsive to LDHA inhibitor therapy (111). These patients typically exhibit more severe disease activity, higher inflammatory burden, and greater joint destruction. Targeting lactate metabolism in this population may provide significant clinical benefits.

Fibrotic phenotype patients may benefit from combination approaches that include anti-fibrotic drugs along with lactate-targeting therapies (112). These patients often have established joint damage and may require more aggressive intervention to prevent further progression.

**Figure 2:**
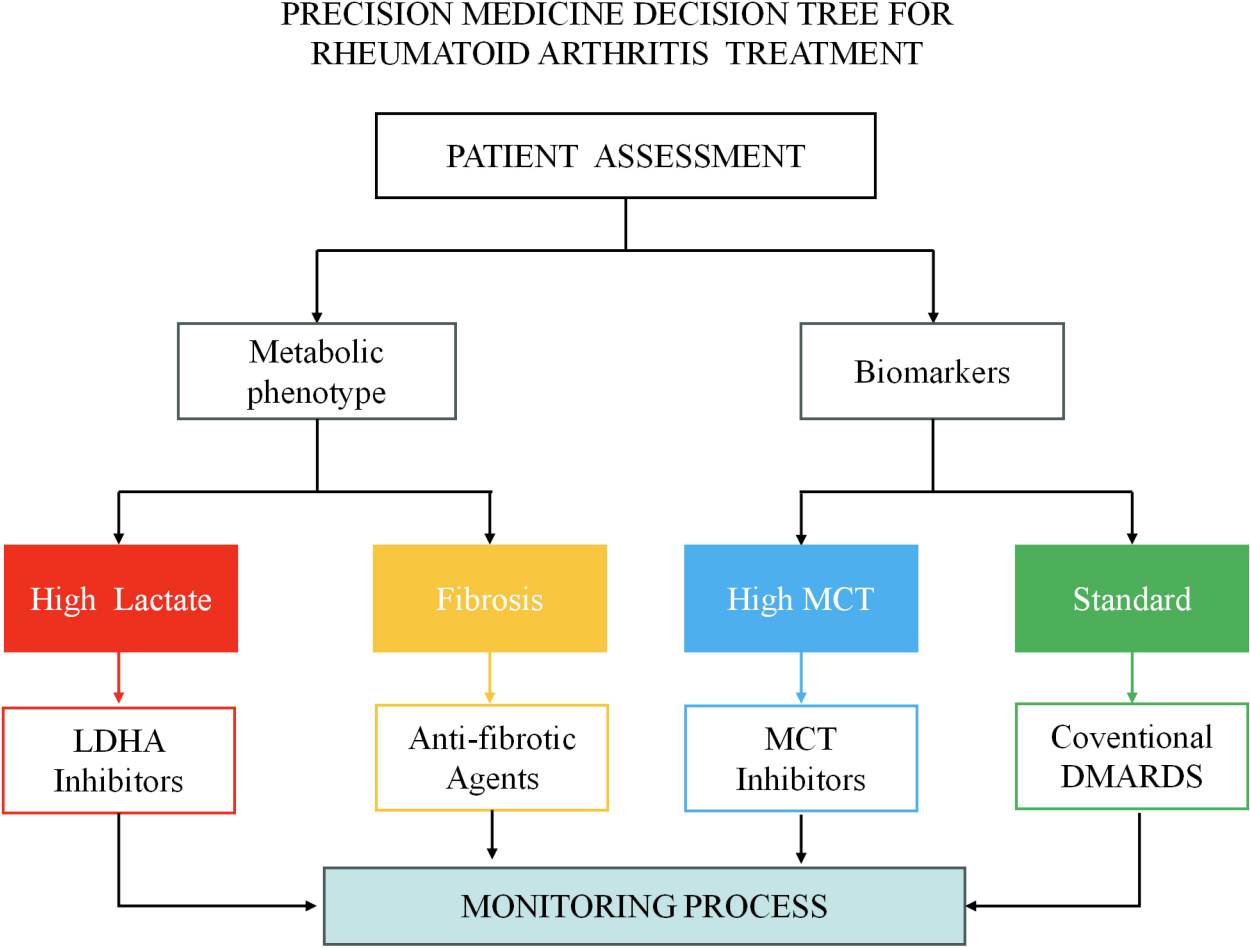
Decision tree for precision treatment of RA based on lactate metabolic biomarkers. The flowchart starts with patient assessment and divides patients into four phenotypes based on serum lactate levels, metabolic phenotypes, and biomarkers: high lactate phenotype (red), fibrosis phenotype (orange), high MCT type (blue), and standard phenotype (green). Each phenotype corresponds to a specific treatment algorithm and monitoring plan to achieve personalized precision treatment.

Lactylation scores derived from proteomic analyses can serve as biomarkers for patient selection and treatment monitoring (113). Patients with high lactylation scores may be more likely to respond to lactylation-modifying therapies, while those with low scores may benefit from alternative approaches. MCT expression levels in synovial tissue can guide the selection of MCT inhibitor therapy (114). Patients with high MCT1 or MCT4 expression may be more likely to respond to MCT-targeting interventions. Genetic polymorphisms in lactate metabolism genes, including LDHA, MCT1, and SIRT1, may influence treatment response and help guide therapeutic selection (115). Pharmacogenomic approaches that integrate these genetic factors may improve treatment outcomes and reduce adverse reactions (**Figure 2**).

### 5.6 Challenges and limitations

Despite the promising prospects for lactate-targeting therapies in RA, several challenges and limitations must be addressed to ensure successful clinical translation (116).

Regarding safety and tolerability concerns, metabolic side effects represent a major concern for lactate-targeting therapies, as lactate metabolism plays important roles in normal physiological processes (117). LDHA inhibitors, in particular, may affect lactate production in tissues that rely heavily on glycolytic metabolism, including skeletal muscle, brain, and heart. Careful dose optimization and patient monitoring are crucial for minimizing these risks.Currently, most of the related candidate drugs are still in the preclinical or early clinical research stage. For example, stiripentol, as an LDHA inhibitor, has a good safety profile in combination with chemotherapy, which brings hope for its clinical application (118). Drug interactions may occur when lactate-targeting therapies are combined with other medications that affect cellular metabolism (119). The long-term safety of chronic metabolic interventions remains largely unknown (120). While short-term studies typically show acceptable safety profiles for lactate-targeting compounds, the effects of long-term treatment on metabolic homeostasis, organ function, and overall health require careful evaluation.

Regarding technical and methodological challenges, drug delivery to synovial tissue remains a significant challenge due to the unique anatomical and physiological properties of joints (121). The synovium, joint capsule, and cartilage matrix can all serve as barriers to drug penetration, potentially limiting the efficacy of systemically administered lactate-targeting therapies. Biomarker validation for lactate-targeting therapies requires the development of standardized assays and reference ranges (122). Additionally, the heterogeneity of RA patients presents challenges for clinical trial design and interpretation. The diverse clinical presentations, disease courses, and treatment responses observed in RA patients may require stratified approaches to clinical development and regulatory approval (123). Finally, it should be emphasized that regulatory pathways for metabolic modulators in autoimmune diseases are still evolving (124). The FDA and other regulatory agencies are developing frameworks for evaluating the safety and efficacy of these novel therapeutic approaches, but the requirements for clinical development may differ from those for traditional anti-inflammatory drugs. The commercial viability of lactate-targeting therapies will depend on their ability to demonstrate clear clinical benefits over existing treatments (125).

## 6 Conclusions

The ultimate goal of developing lactate-targeting therapies is to transform RA from a chronic, progressive disease into a manageable and a condition that can be effectively managed and driven into sustained remission. This vision requires a fundamental shift in treatment approaches from symptom management toward metabolic restoration. The availability of small molecule inhibitors targeting LDHA, MCT transporters, and enzymes that regulate lactylation provides multiple therapeutic entry points for intervention. When these metabolic modulators are combined with conventional biologics, they demonstrate synergistic effects, suggesting that combination therapeutic approaches may achieve superior outcomes compared to either approach alone. The identification of patient subgroups based on metabolic profiles, including high lactate phenotypes and fibrotic variants, enables personalized treatment approaches that optimize therapeutic efficacy while minimizing adverse effects. The development of machine learning-based diagnostic models that incorporate lactylation scores and other metabolic biomarkers provides practical tools for implementing precision medicine approaches in clinical practice.

Looking forward, the integration of lactate metabolism research with emerging technologies such as single-cell metabolomics, spatial analysis, and artificial intelligence promises to accelerate the development of next-generation therapeutic approaches. The vision of personalized metabolic medicine, including real-time monitoring and dynamic treatment optimization, represents the ultimate goal of this research endeavor. Transforming RA from a chronic, progressive disease into a manageable and potentially curable condition requires a fundamental shift in therapeutic philosophy. The goal must be to restore metabolic homeostasis and allow inflammatory processes to naturally resolve, rather than simply suppressing inflammatory symptoms. Targeting lactate metabolism provides a pathway toward achieving this ambitious goal. As we stand at the threshold of the metabolic medicine era, the integration of lactate metabolism research into clinical practice offers unprecedented opportunities for transforming RA from a chronic, progressive disease into a manageable and potentially curable condition.

## Footnotes

1
**Warburg effect: Even under conditions of sufficient oxygen, cancer cells preferentially obtain energy through glycolysis rather than the more efficient aerobic respiration, while producing large amounts of lactic acid, a metabolic feature that provides biosynthetic precursors for their rapid proliferation.**
