# Correction: Targeting lactate metabolism to restore immune homeostasis: a precision therapeutic paradigm for rheumatoid arthritis

**DOI:** 10.3389/fimmu.2026.1802049

**Published:** 2026-02-10

**Authors:** Lin Zheng, Xinyu A, Shiyu Ma, Bo Xu, Zheng Xiang, Boran Cao, Jun Shen, Xirui Xu, Yiwei Cheng, Lei Ran, Qi Shi, Yanqin Bian, Lianbo Xiao

**Affiliations:** 1 Guanghua Hospital Affiliated to Shanghai University of Traditional Chinese Medicine, Shanghai, China; 2 The Research Institute for Joint Diseases, Shanghai Academy of Traditional Chinese Medicine, Shanghai, China; 3 The Department of Pharmacy Ruijin Hospital Affiliated to Shanghai Jiaotong University School of Medicine, Shanghai, China

**Keywords:** lactate metabolism, rheumatoid arthritis, immune homeostasis, precision therapy, lactylation, metabolic reprogramming

An incorrect number was provided for the National Natural Science Foundation of China. The correct number is “(82474302)”.

The funder “the Changning National Master of Traditional Chinese Medicine Studio” was erroneously omitted. The corrected **Funding** statement is:

“The author(s) declare financial support was received for the research and/or publication of this article. This work is supported by the National Natural Science Foundation of China (82474302) and the Scientific Research Project of Changning District Science and Technology Commission (Grant No. CNKW2022Y22, CNKW2024Y19) and was also supported by the Changning National Master of Traditional Chinese Medicine Studio.”

The original version of this article has been updated.

