# Correction: Targeting lactate metabolism to restore immune homeostasis: a precision therapeutic paradigm for rheumatoid arthritis

**DOI:** 10.3389/fimmu.2026.1833571

**Published:** 2026-03-26

**Authors:** Lin Zheng, Xinyu A, Shiyu Ma, Bo Xu, Zheng Xiang, Boran Cao, Jun Shen, Xirui Xu, Yiwei Cheng, Lei Ran, Qi Shi, Yanqin Bian, Lianbo Xiao

**Affiliations:** 1 Guanghua Hospital Affiliated to Shanghai University of Traditional Chinese Medicine, Shanghai, China; 2 The Research Institute for Joint Diseases, Shanghai Academy of Traditional Chinese Medicine, Shanghai, China; 3 The Department of Pharmacy Ruijin Hospital Affiliated to Shanghai Jiaotong University School of Medicine, Shanghai, China

**Keywords:** lactate metabolism, rheumatoid arthritis, immune homeostasis, precision therapy, lactylation, metabolic reprogramming

Author Qi Shi was erroneously omitted as corresponding author. The correct corresponding authors are: “Qi Shi, Yangin Bian and Lianbo Xiao”.

The original version of this article has been updated.

